# P-812. A Guideline-Directed Diagnostic Stewardship Intervention to Reduce Hospital Acquired Infections

**DOI:** 10.1093/ofid/ofaf695.1020

**Published:** 2026-01-11

**Authors:** Ajay K Desai, Ross Taylor, Lavina Davis, Wendi Rittenhouse, Julio Calderin, Olga Karasik, Minh Q Ho

**Affiliations:** University of Central Florida HCA GME, Orlando, FL; HCA Osceola Hospital, Kissimmee, Florida; HCA Florida Osceola Hospital, Auburndale, Florida; HCA Florida Healthcare, Kissimmee, Florida; HCA Osceola Hospital, Kissimmee, Florida; UCF/HCA Healthcare GME, Kissimmee, Florida; Orlando VA Healthcare System, Orlando, FL

## Abstract

**Background:**

Hospital-acquired infections (HAI) are a significant burden to patients, providers, and hospitals, but they may be prevented by properly applying diagnostic stewardship measures to reduce the incidence of catheter-associated urinary tract infection (CAUTI), C difficile infection (CDI), and central line-associated blood infection (CLABSI).

**Figure:**
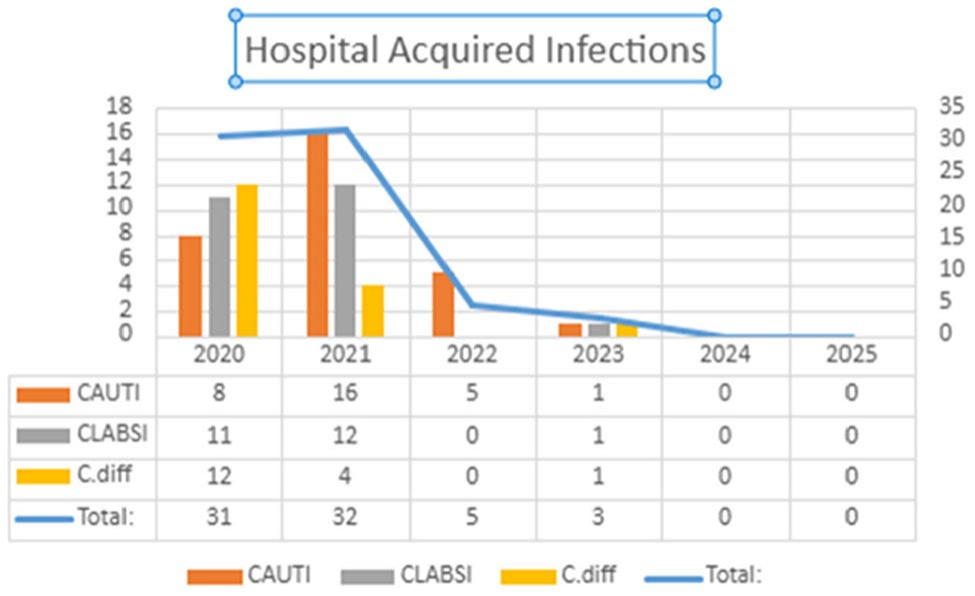
Results depicting the number of HAI broken down to CAUTI, CLABSI and CDI since the implementation of diagnostic stewardship initiatives

**Figure:**
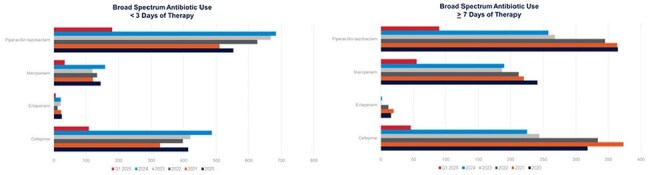
Broad spectrum antibiotic use from 2020-2025, comparing <3 days of therapy and >7 days of therapy

**Methods:**

We describe diagnostic stewardship initiatives at a 404-bed tertiary care Central Florida community hospital to optimize CLABSI, CAUTI, and CDI testing. This initiative is based on 3 overlapping initiatives. The 1st is an electronic medical record alert, which notifies nursing leaders that a test has been ordered that could lead to HAI attribution if positive. The 2nd is using a Diagnostic Checklist (DC) to determine the appropriateness of ordering based on pre-test probability. The CDI DC was implemented in 2020, and the urine and blood DC in 2022. The 3rd intervention involves conversations with ordering providers on whether the test will meaningfully impact treatment. They are asked to consider treatment alternatives that may not involve diagnostic testing if the culture may be responsibly obtained from another anatomic site, if a viable empiric treatment strategy exists, or if the patient is end-of-life and unlikely to benefit from culturing.

**Figure:**
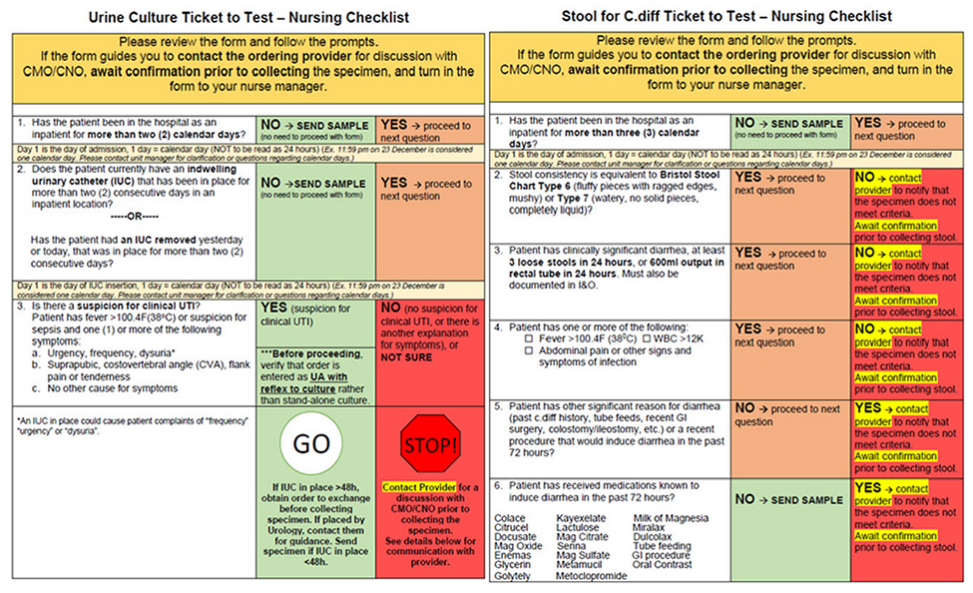
Stool and Urine Checklists Diagnostic checklists implemented for urine cultures and C. Diff stool tests to guide ordering providers

**Figure:**
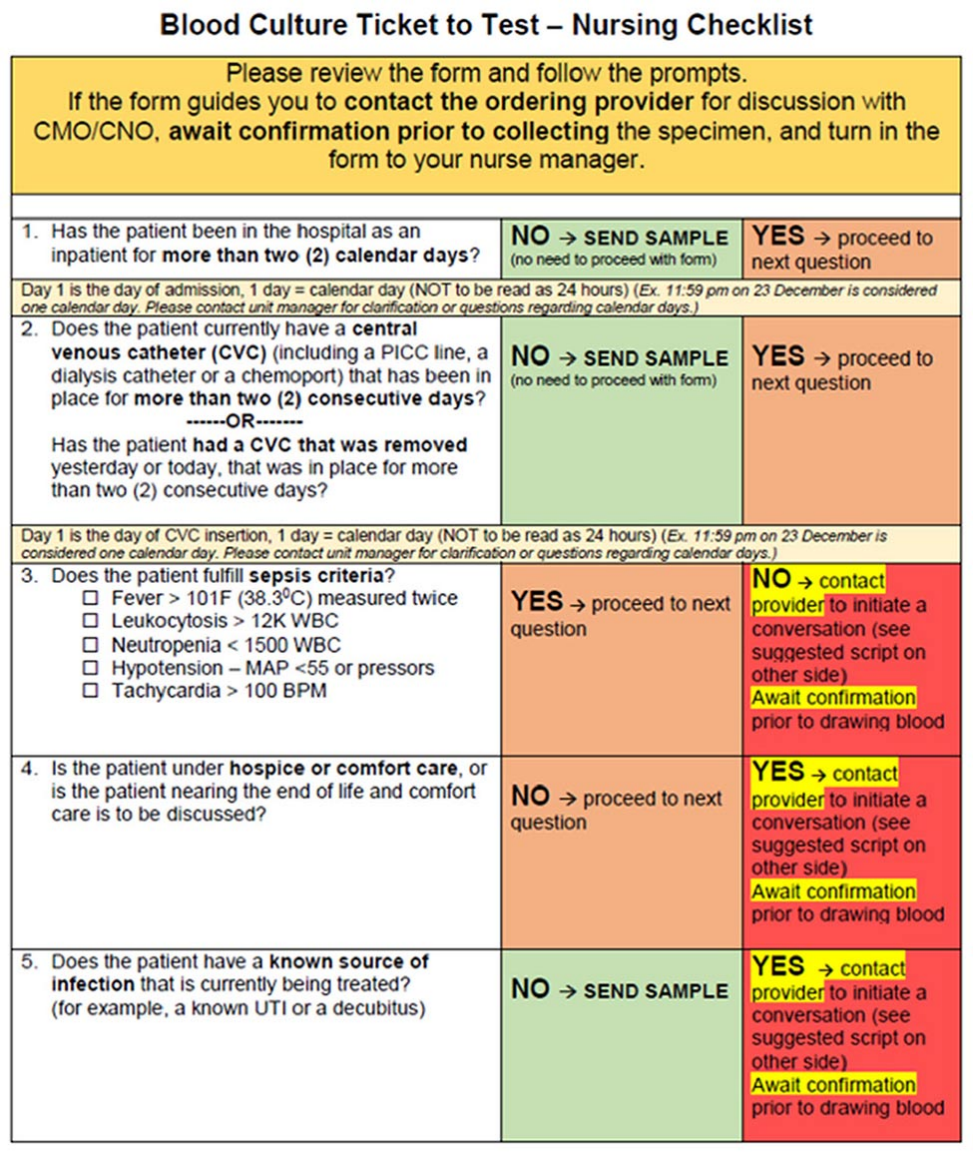
Blood Culture Diagnostic Checklist Diagnostic checklist implemented for blood cultures to guide ordering providers

**Results:**

Since implementation, CDI rate decreased by 66%, and the CLABSI and CAUTI rates declined by 100% and 69% after one year and sustained into 1st quarter of 2025. Providers’ perspective has changed, and they now pause before ordering cultures without needing a DC Checklist. Despite an increase in broad-spectrum antibiotic use of < 3 days, broad-spectrum antibiotic use >7 days has decreased. The key to this initiative's success is the leadership of a Diagnostic Stewardship Workgroup, composed of GME and Medical Staff Leaders and Infectious Disease Physicians. These leaders were key in leading discussions with ordering providers to overcome testing bias inherent to previous generations of learners and providers.

**Conclusion:**

This program is unique for its multidisciplinary collaboration to implement diagnostic stewardship initiatives based on testing alerts, clear criteria, and physician leadership to promote appropriate blood, stool, or urine testing.

**Disclosures:**

All Authors: No reported disclosures

